# Understanding biosecurity behaviors of Australian beef cattle farmers using the ten basic human values framework

**DOI:** 10.3389/fvets.2023.1072929

**Published:** 2023-02-27

**Authors:** Jake Fountain, Jennifer Manyweathers, Victoria J. Brookes, Marta Hernandez-Jover

**Affiliations:** ^1^Gulbali Institute, Charles Sturt University, Wagga Wagga, NSW, Australia; ^2^School of Agricultural, Environmental and Veterinary Sciences, Faculty of Science and Health, Charles Sturt University, Wagga Wagga, NSW, Australia; ^3^Sydney School of Veterinary Science, Faculty of Science, The University of Sydney, Camperdown, NSW, Australia

**Keywords:** biosecurity, value, cattle, behavior, endemic, beef, Australia, COM-B

## Abstract

**Introduction:**

On-farm biosecurity is an essential component of successful disease management in the beef cattle industry on an individual, regional, and national level. Participation in mandatory or voluntary assurance schemes, knowledge and trusted relationships have all been demonstrated to contribute to the development of behaviors that promote biosecurity. However, compliance with rules, socio-psychological relationships and knowledge-seeking behavior are all contingent upon the motivations and beliefs of the individual. It is widely accepted that the motivations and beliefs of all cultures can be defined by ten basic values (Self-direction, Stimulation, Hedonism, Achievement, Power, Security, Conformity, Tradition, Benevolence and Universalism). In this study, we use the ten basic values to characterize the on-farm biosecurity behaviors of Australian beef farmers to facilitate the identification of interventions that are most likely to align with producer motivations and therefore, more likely to result in wider adoption of effective on-farm biosecurity.

**Methods:**

Semi-structured interviews were conducted with 11 Australian beef farmers to discuss the reasons behind decisions to alter or implement biosecurity practices in response to endemic diseases. Thematic analysis was used to identify the motivations, opportunities, and capability of biosecurity behaviors. The ten basic human values were used to characterize these behaviors and inform enablers and barriers to biosecurity adoption.

**Results and discussion:**

Benevolence and Self-direction, relating to self-transcendence and an openness to change, were the principal values associated with good biosecurity behaviors. This suggests that farmers will be receptive to education strategies that communicate the actual risk of disease in their area, the impact of disease on animal welfare, and the ability for on-farm biosecurity to mitigate these impacts. Farmers also expressed values of Security which entrenched behaviors as common practice; however, in some cases the Security of trusted relationships was identified as a potential barrier to behavior change. Overall, values associated with biosecurity behaviors were found to align with values that are most important for social cohesion, suggesting that collaborative disease efforts between industry stakeholders and farmers are likely to succeed if designed with these values in mind.

## 1. Introduction

In the past 2 years, weather events have resulted in restructuring of red meat production systems in Australia. Drought and frequent flood events have resulted in destocking and livestock deaths, causing a decrease in the total number of red meat enterprises (1). Recovery from these events is likely to be accompanied by an increase in the risk of disease transmission as farmers seek to purchase animals to rebuild their herd. Agricultural communities will also be exposed to new challenges due the influence of climate change on the occurrence and distribution of livestock diseases (2). Identification and prevention of new and emerging diseases relies on robust surveillance at all levels of the agricultural supply chain to guide prioritization and response efforts, including at the individual producer-level. However, the size and geography of extensive farming systems, such as those in Australia, can limit the effectiveness of on-farm surveillance, making on-farm biosecurity important for both the individual producer and the agricultural landscape as a whole.

Development and maintenance of an on-farm biosecurity plan in the red meat industries, has been a requirement for accreditation in the Meat and Livestock Australia (MLA) Livestock Production Assurance program since 2017 (3). Whilst this is a voluntary program, lack of participation in livestock assurance can significantly reduce market access and is therefore viewed as a necessity for most commercial Australian red meat producers (4). However, creating a biosecurity plan does not guarantee adherence to a biosecurity plan in practice. While the number of cattle properties with an on-farm biosecurity plan has increased by 65% since 2019, there has been little published evidence that these have resulted in positive behavior changes toward on-farm biosecurity. While they legitimize adherence to a certain standard of biosecurity, mandates and voluntary schemes alone are not enough to assure adherence to positive biosecurity behaviors (5–7).

Ritter et al. (8) and Wright et al. (9) offer foundations to examine the factors that influence adoption of on-farm biosecurity strategies and producer intentions to implement biosecurity. It is evident that a significant number of factors influence biosecurity behaviors, but the factors most mentioned in the literature relate to knowledge. Knowledge of both the disease processes and the benefits of on-farm biosecurity increase the capacity of a producer to prevent disease incursions (7, 8, 10, 11). The source of this knowledge does not need to be formal; it can arise from past experiences with disease incursions or biosecurity measures (7, 10). In the absence of personal experience, a lack of understanding of disease implications might influence a farmer's perception of the efficacy and relevance of on-farm biosecurity which, in turn, reduces willingness to adopt new measures (7, 12). The avenue by which producers obtain information about disease intervention contributes greatly to the perceived efficacy and importance of on-farm biosecurity (7, 13). In Australia, the Farm Biosecurity website (www.farmbiosecurity.com.au) provides a good foundation for cattle farmers to understand and improve on-farm biosecurity. Some Australian state government websites also provide resources specific to beef cattle farmers (14–16) and there are several voluntary programs designed to educate and incentivize biosecurity behaviors (17, 18). With plenty of resources designed to educate Australian farmers about the benefits of biosecurity, lack of availability of information should not be a limiting factor. However, copious amounts of information can be excessive, contradictory and might not align with the information-seeking pathways of cattle farmers (19). In a profession for which time is a valuable commodity, famers often circumvent the need to sift through information by relying on communication with trusted stakeholders in their industry (7, 20, 21).

Farmers are most likely to act on information provided by trusted advisors, and the influence of a trusted veterinary practitioner on farmers' decision-making should not be understated (8, 10). Private veterinarians are consistently regarded as the most important source of knowledge and advice on biosecurity, regardless of enterprise type or country (7, 19, 21–23). However, cost and geographic distance from veterinary services can be a barrier to veterinary knowledge, especially for extensive production systems such as those in Australia (9, 24). Even when veterinary advice is readily accessible, farmers might be reluctant to discuss on-farm biosecurity if veterinarians appear apathetic when compared to other aspect of cattle health (23). Also, a farmer who perceives a veterinarian's salient values as antithetical to their own may lead them to seek advice from others in the farming community (9). Rural social networks are used by farmers to address knowledge gaps left following private veterinary interactions (25). Discussions between neighboring producers have been identified as a primary source of information for farmers, regardless of enterprise type or size. Some producers, especially Australian smallholders, also take advice from those with whom they hold a close personal relationship, such as family or friends (8, 20).

It is important to acknowledge that the socio-psychological relationship between a trusted advisor and farmer, knowledge-seeking behavior, and receptiveness to changes, are all contingent on the personality, attitudes and beliefs of an individual producer (8). Additionally, many of the other factors expressed in the literature that influence biosecurity behaviors, such as a producer's perception of risk and responsibility toward prevention of disease, are also shaped by individual beliefs and motivations, as described by value-belief norms theory (9, 22, 26). Gorddard et al. (5) further describe “values” as the system which articulates the purpose of a decision and describes their interdependent relationship with knowledge and rules. It can be argued that farmers are not a homogenous group and thus, we should seek to understand individual belief-systems and avoid conflict with these values when attempting to guide farmers toward a change in disease management (22, 27). Accomplishing this for each individual producer would require a vast amount of time and personnel in an industry which is already resource deficient. However, socio-epidemiology acknowledges that farmers' individual belief systems and behaviors are shaped by social norms (i.e., descriptive^1^ and injunctive norms^2^) which stem from a set of shared cultural values. This social cohesion, when interpreted collectively with moral values, might justify judicious inference of shared values between producers of similar typologies (8, 20). Approaches such as Burton and Wilson's (28) description of “productivist,” “post-productivist” and “multifunctional agricultural” regime of farming typology, whist not tailored to the individual, might be used to increase the impact of extension programs that adhere to the underlying values of the beef farming community.

**Figure 1:**
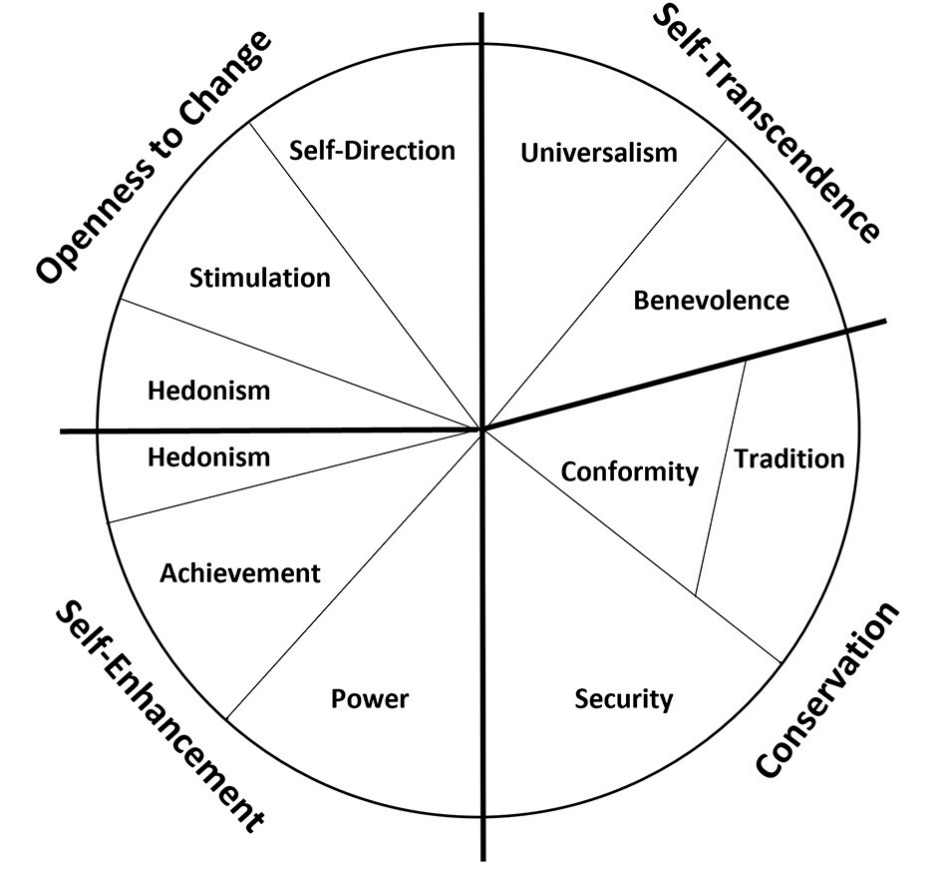
Theoretical model of relationships between the ten motivation types of held value [obtained from Schwartz (29). CC BY-SA 4.0]. The more distant the two values on the continuum, the more antagonistic their motivations. Tradition and conformity are located in a single wedge as they share the same broad motivational goal; the absolute nature of tradition values demanding a stronger rejection of opposing values. Solid lines denote the value's relationship with the respective motivational dimension.

“Values” can be characterized in several ways; but perhaps the most well recognized representation are the ten basic values described by Schwartz (29): Self-direction, Stimulation, Hedonism, Achievement, Power, Security, Conformity, Tradition, Benevolence and Universalism. Every person employs a combination of these values to any motivational situation. Schwartz (29) explains that values transcend specific actions and situations, which makes them applicable across various decision contexts. Schwartz (29) goes on to describe the conflict-congruency relationship between the values, outlining the bipolar dimensions: “openness to change” vs. “conservation” and “self-enhancement” vs. “self-transcendence” (Figure 1). The relative importance of values and their intrinsic competition with each other guides attitudes and behavior. The ten basic values have not been applied to agricultural decision behaviors. In the context of beef farming, most studies based in Australia have collected knowledge, attitudes and behaviors of beef producers, with little focus on the underlying process of decision-making behavior (12, 30–32). Higgins et al. (33) explore and describe the relationship between producer “practices of care” and established “biosecurity principles” for the Australian beef industry, drawing on the internal process of biosecurity behaviors. More recently, Michie et al. (34) capability, opportunity, motivation behavior (COM-B) system has been used to understand the psychology of BVDV control in British cattle farmers (35). Briefly, the COM-B system describes how an individual's behavior is characterized by their (psychological and physical) capacity to engage in a behavior, the (physical and social) opportunities that prompt a behavior, and the (reflective and autonomic) motivation to energize the behavior. Scrutiny of the COM-B system has revealed that it accounts for a wide range of the broad theories from the behavior literature (36), but perhaps most importantly for this current study, reflective and autonomic motivations are inherently linked to Schwartz's (29) values.

This study builds on the current literature by using the ten basic values to characterize the on-farm biosecurity behaviors of Australian beef farmers. Framing biosecurity behaviors in value theory will facilitate the identification of practices that best align with producer motivations and thus are more likely to result in wider adoption of effective on-farm biosecurity. Values will be extrapolated primarily from aspects of management decisions relating to, but not limited to, prevention of infectious endemic diseases, because there is a perception amongst producers that the government is primarily responsible for the management of emerging diseases, and it is generally accepted that farmers perceive an endemic disease incursion as more relevant to their production system (19, 21, 30).

## 2. Materials and methods

### 2.1. Participant recruitment

This study builds on previous studies exploring the values that shape the biosecurity behaviors of Australian beef producers (37, 38). The recruitment and data collection process for this study were approved by the Human Research Ethics Committee of Charles Sturt University (protocol number H21370) and an example of the participant information statement provided to all participants can be found in the Supplementary material. Beef producers were recruited for interviews from an online survey to obtain producer preferences about prevention of endemic diseases (38), in which any landholder who farmed a beef cattle herd of any size in Australia could participate with the option to volunteer for a follow-up interview by providing their contact information at the end of the survey. Only interview participants who submitted a completed survey were considered. Eleven participants were selected from the eligible candidates based on location (state), herd size, beef farming experience, and availability to participate, to ensure a diverse population of responses.

### 2.2. Data collection

Semi-structured interviews with 11 Australian beef farmers (including one pilot interview) were conducted, with a duration of 60–90 min for each interview (see Supplementary material for interview script). All interviews were conducted by the primary author over Zoom video conferencing software (www.zoom.us) or telephone, based on participant request. The interviews were digitally recorded for transcription. The focus of the interviews was to discuss reasons behind historical decisions to alter or implement biosecurity practices in response to endemic diseases in their beef herd. Therefore, participants were also asked for their definition of an endemic disease to put their responses into context and ensure a mutual understanding of the intended discussion points of the interview. We also acknowledge that the implementation of biosecurity practices for endemic disease prevention can be viewed as a holistic part of farm animal health management (33) and so discussions raised by the participant about the decision-making process of any aspect of farm animal health was permitted. We also used the participant preference scores from Fountain et al. (38) to promote further discussion about the use of specific biosecurity practices. A short self-reflection on the process was conducted using a voice recorder by the interviewer immediately after each interview to provide commentary and context to the reader.

### 2.3. Data analysis

The interview transcripts were analyzed in NVivo Pro 12 (39). A mixture of deductive and open coding was used to identify and describe concepts relating to the participants' understanding of infectious endemic diseases, reasons for pursuing a career in beef cattle production and use of on-farm biosecurity practices. Thematic analysis was used to identify recurring themes describing the motivations, opportunity, and capability of farming decisions. The COM-B system was used to compartmentalize and provide context for the farming decisions identified during thematic coding. Motivations, opportunities, and capabilities were then scrutinized to develop a “values profile” for each farming practice.

The ten basic human values were applied to farming behaviors by qualitative judgements derived from the authors' interpretation of the defining goals and motivational types from Schwartz (29) and Schwartz (40). The authors of this study were not producers or primary caregivers of production animals but have been involved in a variety of animal care backgrounds including mixed veterinary practice, veterinary epidemiology, and academia. The team had combined experience with semi-qualitative and qualitative research regarding behaviors and practices of small and large-scale producers in the Australian agricultural landscape. Triangulation occurred throughout the analysis to ensure that the authors' lens captured the nuances regarding the drivers of producer behavior; however, should be noted that this interpretation might differ from those with lived on-farm experience.

Value-allocations were routinely scrutinized throughout the analysis to ensure consistency and transparency when describing the values attributed to the various biosecurity behaviors. For example, Benevolence and Universalism contain overlapping themes relating to welfare; however, Benevolence was attributed to motivations relating to the welfare of agents within the farmer's network, while Universalism related to preserving the welfare of the greater farming community and the natural environment. Security, Conformity and Tradition also contain overlapping themes of stability and social conservation. The authors interpreted Security-related behaviors as those that foster a sense of belonging or self-comfort, while Conformity was attributed to behaviors that satisfy the requirements of others to maintain social relationships. Tradition, on the other hand, described strict, unwavering adherence to behaviors at all costs, with no intention to change or alter the behavior when circumstances change. Table 1 provides a brief description of all the value concepts used in this study and how they relate to biosecurity behaviors. To avoid researcher-derived assumptions relating the value-attributes, values were only assigned to behaviors if the motivation was explicitly mentioned by participants during the interview process.

**Table 1:** Description of the ten basic values (in order of cultural importance) as well as author interpretation of values in relation to farming behaviors.

**Value**	**Defining goal^a^**	**Primary motivational type^b^**	**Farmer-specific attributes^c^**
**Benevolence**	Preserving and enhancing the welfare of those with whom one is in frequent personal contact	• Helpful • Loyal • Forgiving • Honest • Responsible • True friendship • Mature love	• Animal welfare • Responsibility for employees • Responsibility for supply chains
**Universalism**	Understanding, appreciation, tolerance, and protection for the welfare of all people and for nature	• Broad-minded • Social justice • Equality • World at peace • World of beauty • Unity with nature • Wisdom • Protecting the environment	• Environmental concern • Climate change • Antimicrobial resistance • Conservation • Biodiversity
**Self-direction**	Independent thought and action – choosing, creating, exploring	• Creativity • Freedom • Choosing own goals • Curious • Independent	• Financial independence • Self-employed • Self-sufficient • Confidence • Self-improvement • Insistent • Proactive • Innovative • Information-seeking • Deviation from norms
**Security**	Safety, harmony, and stability of society, relationships, and self	• Social order • Family security • National security • Reciprocation of favors • Clean • Sense of belonging • Healthy	• Maintaining relationships • Consistent/Routine behavior • Trust • Social cohesion • Participation in schemes • Cooperation
**Conformity**	Restraint of actions, inclinations, and impulses likely to upset or harm others and violate social expectations or norms	• Obedient • Self-discipline • Politeness • Honoring parents and elders	• Adherence to norms (family, social, etc.) • Avoiding confrontation • Politeness
**Hedonism**	Pleasure of sensuous gratification for oneself	• Pleasure • Enjoying life	• Enjoyment • Lifestyle choice • Passion
**Achievement**	Personal success through demonstrating competence according to social standards	• Ambitious • Successful • Capable • Influential	• Success • Genetic improvement • Pride • Competence • Productivity
**Tradition**	Respect, commitment, and acceptance of the customs and ideas that one's culture or religion provides	• Respect for tradition • Humble • Devout • Accepting my portion in life • Moderate	NA
**Stimulation**	Excitement, novelty, and challenge in life	• A varied life • An exciting life • Daring	• Interest in farming
**Power**	Social status and prestige, control or dominance over people and resources	• Authority • Wealth • Social power • Preserving public image • Social recognition	NA

a Sourced from Schwartz (29).

b Sourced from Schwartz (40).

c Author-derived interpretations. NA, Not applicable/not identified.

## 3. Results

Overall, participants were interested, and all were willing to engage in the interviews. The initial question regarding the definition of “infectious endemic disease” was received with some apprehension; however, it did succeed in promoting a greater understanding of the interview context. All subsequent discussion was conducted as a general conversation rather than a strict “question and answer”, providing a narrative on discussed topics. When asked about their experiences with “endemic” diseases, most participants were willing to provide detailed historical accounts and extrapolated on specific decisions when prompted. The questions and conversation were directed by the participant, while the interviewer asked for elaboration to ensured that enough detail was obtained to gather the key information required to satisfy the objectives of the study. This approach reduced discomfort between participants and interviewer and contributed to rapport and trust between parties.

### 3.1. Description of participants

**Figure 2:**
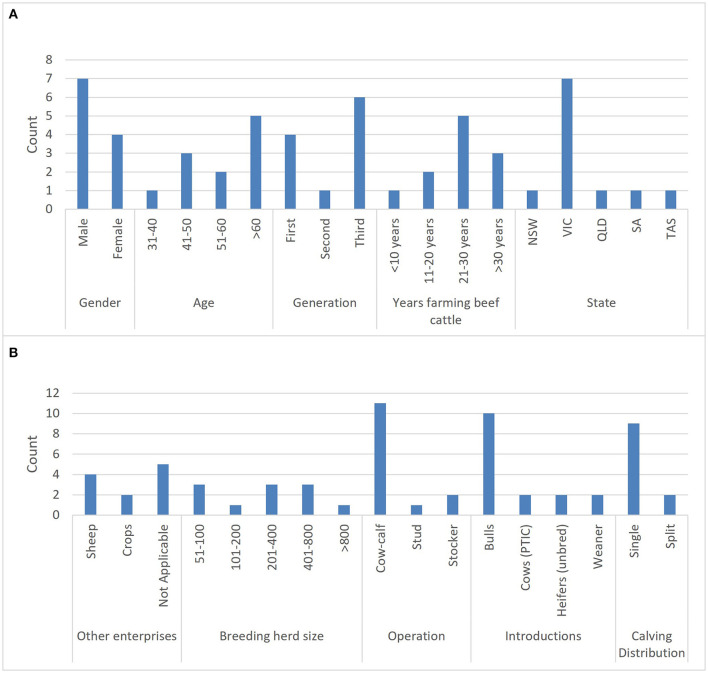
Barplots illustrating counts of the demographics **(A)** and enterprise details **(B)** of Australian farmers participating in semi-qualitative interviews. NSW, New South Wales; VIC, Victoria; QLD, Queensland; SA, South Australia; TAS, Tasmania; PTIC, Pregnancy tested in-calf.

Of the 11 interview participants, seven identified as male and four identified as female (Figure 2). All female participants were between 40 and 50 years old and of these participants, three were third generation^3^ beef farmers and one was a first generation^4^ beef farmer. Of the male participants, three were first generation beef farmers >60 years old, one was a second generation^5^ beef farmer >60 years old and three were third generation beef farmers of 31–40, 41–50, and >60 years old. Seven out of 11 participants farm in the state of Victoria. Five participants farmed only beef cattle, four farmed sheep alongside beef cattle and two participants ran a cropping enterprise alongside their beef operation. Two of the participants in this study were part of the same farming operation and one participant had very recently retired from beef cattle farming.

### 3.2. Defining infectious endemic disease

When asked to define “infectious endemic disease,” most participants broke the definition down into two parts: “infectious” and “endemic.” Most farmers (*n* = 8) provided a response that aligned with the researchers' interpretation of “infectious disease” with two participants not answering the question sufficiently to make a judgement and one indicating that an infectious disease is caused by “*bugs that the animals have caught on the farm… could be footrot” (Participant 2)*.

Four of 11 participants described ‘endemic disease' in a way that conformed with the researchers' expectations. The following quotes provide some examples of the predominant interpretation of an ‘endemic' disease for those producers with a different interpretation: “*a disease in a particular place” (Participant 3), “they're the ones that are around forever” (Participant 6), “it's just there” (Participant 7)*, and “*detrimental to the health of the whole herd” (Participant 11)*. Some participants mentioned opportunistic diseases such as pneumonia as examples of endemic diseases, while two producers mentioned exotic diseases such as lumpy skin disease, brucellosis, and tuberculosis. One participant suggested that categorizing every cattle disease into endemic and non-endemic might be a superfluous exercise:

“*I guess for me it's a question not so much about what tag you attach to the issue. It's what the issue is, and how you deal with it. Whether you call it endemic, or whether you call it something else.”*

–* Participant 4*

**Figure 3:**
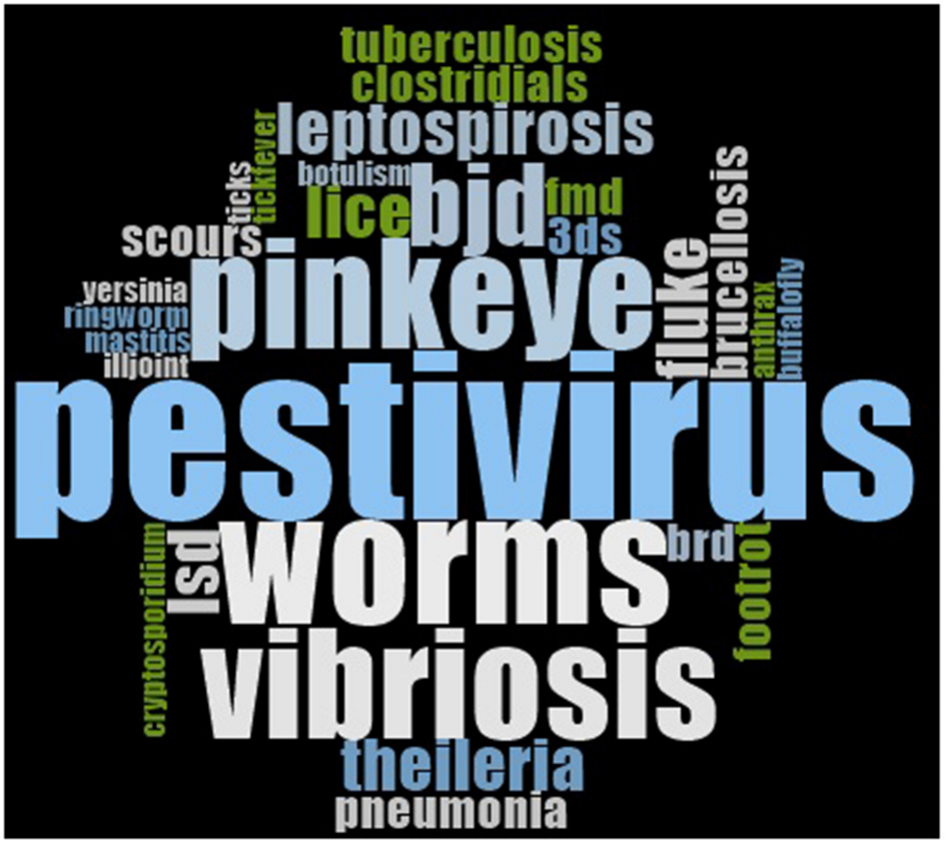
Word cloud illustrating the diseases mentioned by participants during qualitative interviews discussing the prevention of endemic diseases in Australia. BJD, Bovine Johne's disease; LSD, lumpy skin disease; 3ds, three-day-sickness; BRD, bovine respiratory disease; FMD, foot-and-mouth disease.

Throughout the interviews, all participants mentioned pestivirus (bovine viral diarrhea virus, BVDV; *n* = 11) as a disease of interest (Figure 3). More than half of the participants also mentioned worms (internal parasites; *n* = 8), pinkeye (*n* = 7) and vibriosis (campylobacteriosis; *n* = 7). Less common endemic diseases mentioned by the participants included Bovine Johne's disease (BJD; *n* = 5), liver fluke (*n* = 3) and cattle lice (*n* = 3). Region-specific endemic diseases were also mentioned in the interviews, including Theileria (*n* = 3), 3-day-sickness (*n* = 2), ticks (*n* = 1) and associated tick fever (*n* = 1).

There was a temporal factor associated with the interest that participants directed toward specific diseases, with several participants indicating recent experiences and current events as the main contributor. One participant also indicated that frequent discussions regarding endemic diseases are an important to prompt reassessment disease importance in relation to their enterprise:

“*Talking about pestivirus in particular, it's a really good thing to be doing because it's not the sort of thing that is front of mind – coincidently it feels like it's been about a year since I last talked about it with my vet and now talking to you has brought it up in my mind again to critically check what we're doing is still the right thing.”*

*Participant 8*

### 3.3. The decision to farm beef cattle

**Figure 4:**
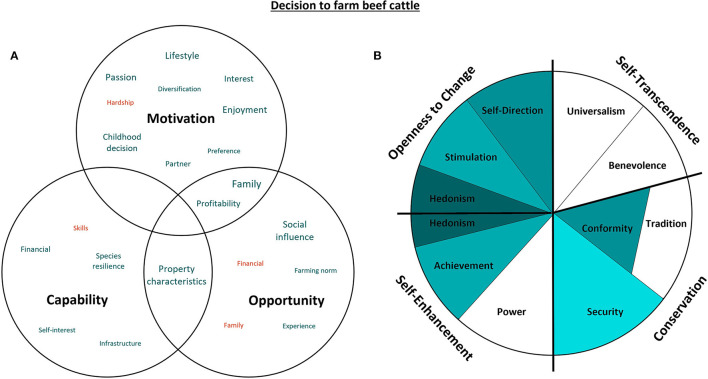
Capability, opportunity, motivations and values associated with the decision to farm beef cattle. Green represents attributes that support the decision to farm beef cattle, while red indicates confounding attributes. The size of the text in the Venn diagram **(A)** indicates the commonality of themes amongst the participants, as does darker shading in the value wheel **(B)** [adapted from Schwartz (29). CC BY-SA 4.0].

Figure 4 represents the capability, opportunity, motivations and values associated with the decision to farm beef cattle.

Hedonistic and Stimulation values relating to enjoyment, passion, work-life balance, and a general interest were the predominant themes associated with farming beef cattle. The characteristics of the property and the resilience of cattle as a species afforded many participants with both the opportunity and capability to work with cattle on a part-time basis, which complemented the Hedonistic values relating to enjoyment and work-life balance:

“*[I farm beef cattle] for the enjoyment and reward of producing good animals.”*

–* Participant 2*

“*We believe the country that we now run is very suited to beef cattle.”*

–* Participant 5*

“*It's more manageable… cows are a little bit more forgiving in terms of the amount of husbandry one needs to provide, compared to sheep.”*

–* Participant 10*

For those producers who did not come from a farming background, goal-oriented and financially independent themes relating to Self-direction facilitated the acquisition of farming land and cattle. Most of these participants had previously been employed in financially stable roles both within and outside of the agricultural sector (veterinarian, stock-and-station agent, pharmaceutical representative, agricultural journalist, public servant, research, tertiary administration, property investor, gardening). Profitability and the ability to improve the farming enterprise were themes relating to Achievement values that provided both the opportunity and motivation for these producers to remain in the beef industry. Hedonism, Self-Direction, Stimulation and Achievement are personal values associated with an openness to change. This suggests that producers might choose to leave the industry in times of hardship (such as those that experience drought or natural disaster) or might retire due to degradation in skills and abilities:

“*I retired because I can't move quickly enough to get out of the way of a steer moving toward me anymore.”*

–* Participant 1*

Egress from the farming industry can also be accompanied by handing down the enterprise to successors, which was the major motivation for commencing or continuing beef farming amongst the interview participants. Most grew up farming cattle, which fostered a sense of belonging within the sector related to Security values. The Security of family operations provided some participants the comfort of pursuing other careers with the knowledge that they could re-enter the beef farming industry at any time:

“*I spent 15 years as an agronomist, [but came back to cattle farming because of] family – I really liked that [agronomy] job working with other farms, but this is always where I was going to end up.”*

–* Participant 8*

Even when producers didn't grow up with cattle, “living off the land” was a big enough motivator to consider farming later in life, stemming from the Security of a childhood decision to enter the farming industry. When producers did not grow up farming cattle, Security values associated with a supportive social environment cultivated the motivation and capacity to enter a beef cattle farming role:

“*Both my parents and my wife's parents came off the land, so we had some connection with farming, intrinsically.”*

–* Participant 10*

“*When I was at school, I wanted to be a farmer. I had an interest in biology and we had an extremely good biology teacher who triggered my interest.”*

–* Participant 1*

Conformity values contributed to the decision to farm beef cattle if participants were not from a regional or farming background. Reliance on advice from neighbors in the farming community resulted in adoption of cattle farming as a norm within the region. As adjacent values on the value-wheel (Figure 4B), these Conformist themes overlap with the Security values with a shared motivational emphasis to protect the harmony between relations:

“*We were really just encouraged by our neighbors to give beef cattle a go and we really enjoyed it.”*

–* Participant 3*

### 3.4. On-farm biosecurity practices

#### 3.4.1. Isolating cattle

**Figure 5:**
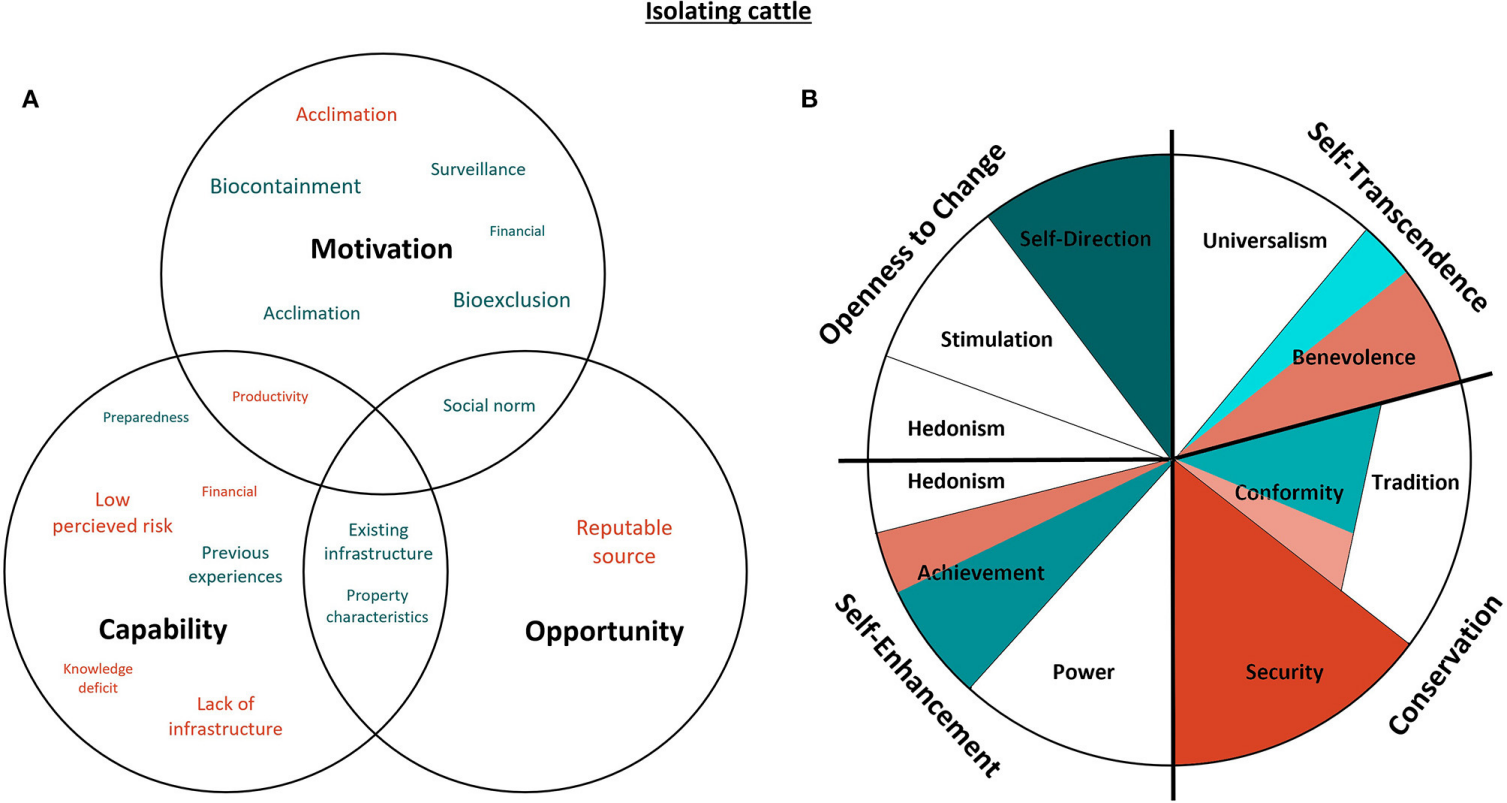
Capability, opportunity, motivations and values associated with isolation of introduced cattle. Green represents attributes that promote the desired behavior, while red indicates confounding attributes. The size of the text in **(A)** indicates the commonality of themes amongst the participants, as does darker shading in **(B)** [adapted from Schwartz (29). CC BY-SA 4.0]. Values with both green and red shading both promote and confound desired isolation behaviors.

There were two main interpretations of “isolation” by the interview participants: isolation from the entire herd for management of disease (*n* = 10) and isolation from all but a portion of the herd for acclimation to the farm (*n* = 6). For the purposes of this study, complete isolation was the “desired” biosecurity behavior to prevent disease transmission and so isolation for the sole purpose of acclimation was considered to compromise adequate isolation of introduced cattle. Figure 5 represents the capability, opportunity, motivations and values associated with isolation of introduced animals.

Self-direction was the predominant value associated with the researchers' “desired” isolation behaviors, relating to motivations that prevent disease introduction (bioexclusion) and disease spread within the herd (biocontainment). Bioexclusion was referenced particularly when discussing the introduction of breeding females. The motivation to prevent disease transmission was attributed to proactive behaviors, producer self-confidence in their ability to prevent disease, self-assessment of risk, and historical experiences that resulted in deviation from previous isolation behaviors. Participants also indicated that isolating cattle afforded the opportunity for enhanced disease surveillance and protection of financial investments:

“*We segregate the young bulls; particularly given the price you've got to pay for them at the moment.”*

–* Participant 10*

Conversely, Security values were most antithetical to the researchers' “desired” isolation behaviors. Existing relationships between supplier and producer contributed to a low perceived risk of disease introduction, which negated the perceived requirement for complete isolation of purchased cattle from the rest of the herd. These relationships were also important to maintain productivity, wherein the isolation practices are forgone for the sake of reproductive success:

“*Because we want to have our heifers in calf within that joining period – I will ring the stud, they will have a replacement bull delivered – the injured bull will come out and the replacement bull will go in.”*

–* Participant 7*

This serves as an example of how Achievement values can negate isolation behavior. However, producers with a focus on productive farming outputs were also more likely to practice rotational grazing, and this existing infrastructure (separate grazing paddocks) would facilitate in the isolation of introduced or clinically sick cattle. There was a sense of pride in the acquisition of multiple properties and the ability to purchase cattle earlier than required, which is also reflective of Achievement values.

Benevolent values relating the welfare of the existing beef herd was identified as a reason for strict isolation of introduce or sick cattle; however, Benevolence was primarily associated with isolation for acclimation of introduced cattle, which is contrary to the researchers' “desired” isolation behavior. It should be noted that acclimation of individual animals might also convey benefits to on-farm productivity and hence relate to Achievement values; however, this was not a motivational theme identified in the interviews. Isolation practices for the purpose of acclimation were implicitly related to animal welfare, where purchased bulls would be introduced to established bulls to maintain social hierarchy prior to joining, or to late-calving cows to facilitate acclimation of the rumen microbiome and the bull's immune system:

“*You introduce them to older bulls or a few dry cows – to help the rumen bugs cross contaminate – and get them used to the bugs on our place.”*

– *Participant 5*

Conformity values were related to isolation behaviors for producers who were new to beef farming. Isolation behaviors were found to conform with those “desired” by the researchers when adhering to social norms regarding “good farming practices.” However, reliance on assistance from neighboring producers in the early stages of a farming enterprise could also contradict desired isolation behaviors for the sake of politeness:

“*Back then we were still cutting our teeth a little bit – [our neighbor] would bring [the bulls] in and we would just put them straight into the paddock with the cows.”*

–* Participant 4*

#### 3.4.2. Protecting farm boundaries

**Figure 6:**
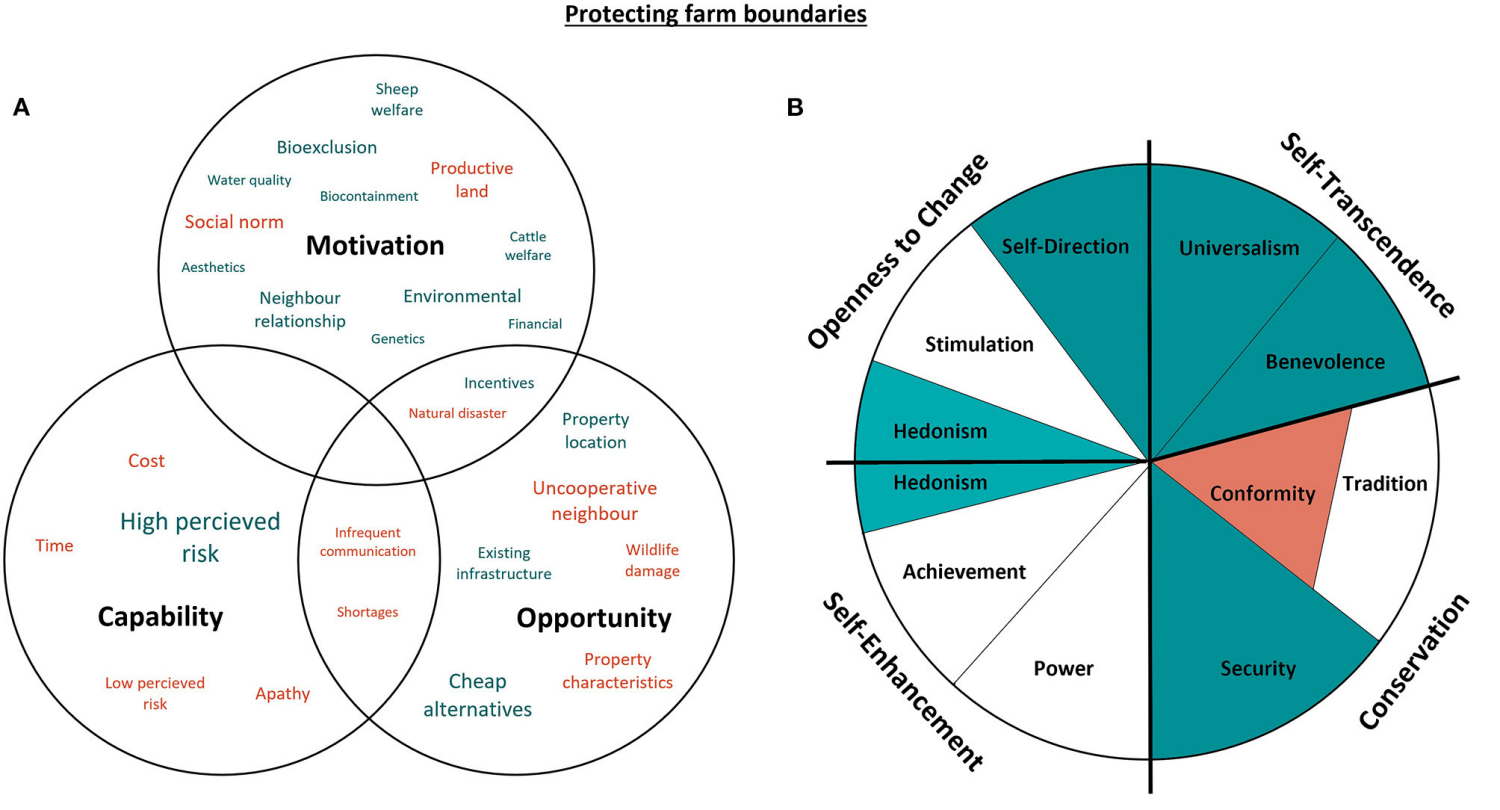
Capability, opportunity, motivations and values associated with protecting farm boundaries. Green represents attributes that promote the boundary protection, while red indicates confounding attributes. The size of the text in **(A)** indicates the commonality of themes amongst the participants, as does darker shading in **(B)** [adapted from Schwartz (29). CC BY-SA 4.0].

When discussing protection of farm boundaries, this referred mainly to the use of double fencing and shelter belts (tree lines planted between double fenced areas). Figure 6 represents the capability, opportunity, motivations and values associated with protecting farm boundaries.

Universalism, Benevolence, Security and Self-direction values were most associated with the protection of farm boundaries. Universalist and Benevolent values are related to the enhancement of others and transcendence of selfish interests. These themes were relevant specifically to the use of shelter belts, which were indicated by participants to improve biodiversity and reduce the impact of adverse weather conditions on livestock. The latter Benevolent theme was particularly prevalent amongst those cattle producers who also farm sheep, being more likely to recognize the protective benefits. Hedonist values were also a theme related to the use of shelter belts *via* enjoyment of the aesthetic improvements that they afford to the environmental landscape:

“*It's something I really enjoy – because we get a lot of birds and I quite like mustering up and down the lanes, looking at the different parrots that we've got coming and going.”*

–* Participant 6*

Universalism, Benevolence and Security are values with a social focus, which suggests that the benefits of shelter belts extend beyond the individual farm. Security values were expressed through the importance of maintaining relationships with neighboring farmers, specifically relating to the mutual protection of both farming enterprises from disease and genetic mixing:

“*Good fences make good neighbors. The best thing you can do is have really good fences between you and your neighbors to minimize issues.”*

–* Participant 7*

The prevention of disease entry onto farm (bioexclusion) was the most cited motivation for protecting farm boundaries. Some participants chose not to pursue reinforcement of boundaries to avoid insulting their neighbors, stemming from the Conformist values associated with politeness. However, the predominant value associated with the decision to protect farm boundaries was Self-direction. Farmers were more likely to carry out the practice on their property if they perceived the over-the-fence transmission risk to be high:

“*Most of our neighbors are fairly – like, the beef guys we're not concerned about. The couple of guys that run the dirty dairy stock are a little bit more concerning…”*

–* Participant 8*

The inverse relationship between Self-direction and Conformist values highlights the importance of the neighboring producer. Neighbors who were uncooperative were identified as the biggest barrier to implementation of double fencing. Participants indicated that financial cost and time were the predominant factors precluding the erection of double fences and therefore, there is an expectation that neighboring farmers should be partially responsible for construction. In response to apathetic or uncooperative neighbors, participants who value Self-direction over Conformity were more likely to adopt cheaper alternatives, such as an offset hotwire, or alter the grazing system as a partial solution to protecting farm boundaries:

“*Over the years I've had different neighbors and some neighbors have been dairy – so most of my fences are electrified to reduce transmission over the fence.”*

–* Participant 2*

These partial solutions also addressed the principal argument against the use of shelter belts, namely the loss of productive land for available grazing. One producer also indicated that shelter belts, left unattended, might make the farm prone to the impact of bushfires. Frequent flooding and property topography were factors that reduced opportunity for implementation of double fencing, due to the position of water courses or the distance of the fence line in more extensive operations. Alternatively, properties located next to natural barriers such as roads or bodies of water were acknowledged as facilitators of farm boundary protection, as well as the presence of already established double fences on purchased or inherited land. One producer indicated that government or industry incentives to improve fencing would negate many of the barriers that make producers reluctant to undertake double fencing:

“*If the government came around and said: ‘we're going to put up a double fence for you,' I would think you'd find 90% of farmers would say ‘yep.”'*

–* Participant 2*

#### 3.4.3. Fomite control

**Figure 7:**
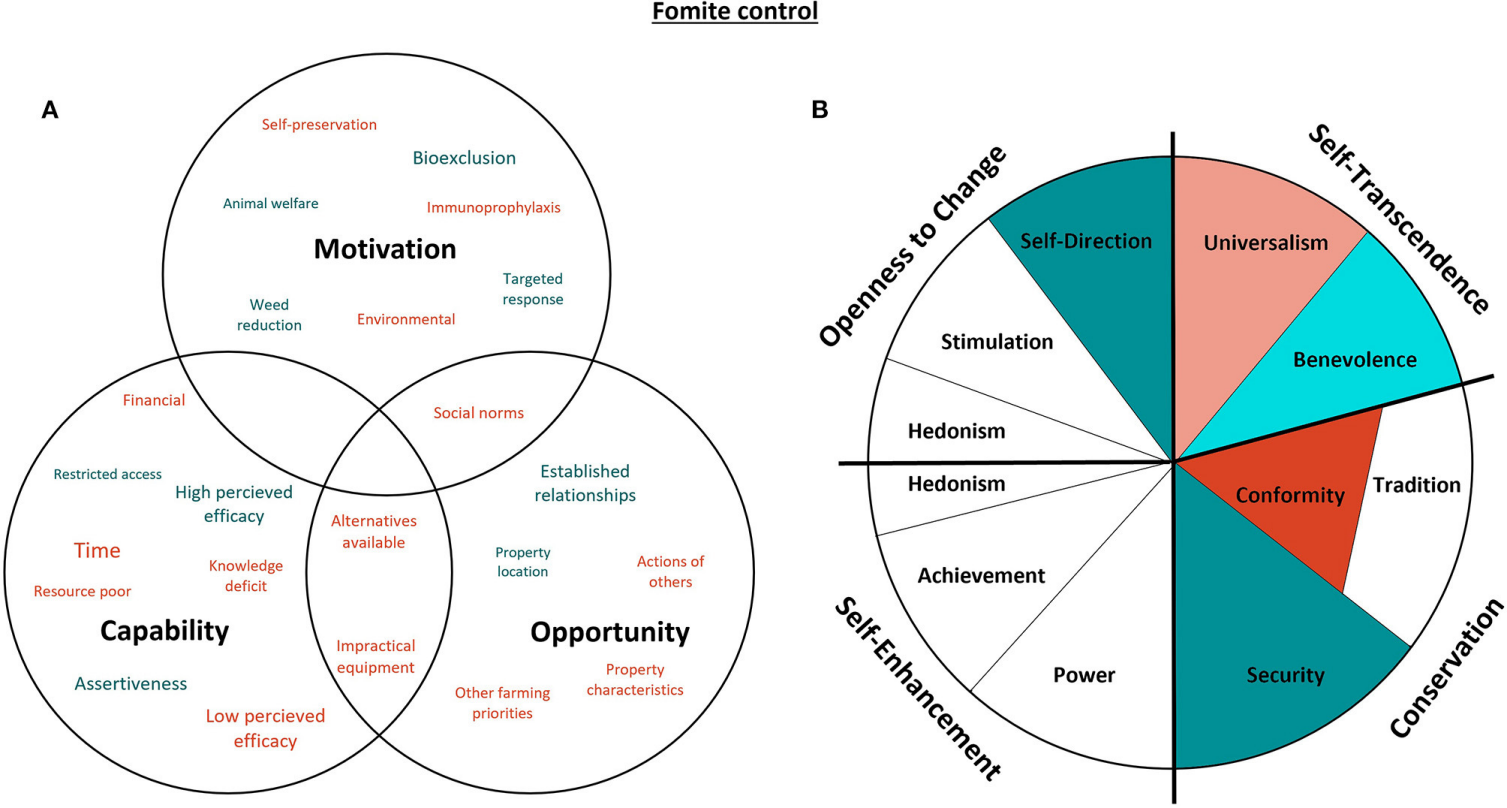
Capability, opportunity, motivations and values associated with the control of fomites. Green represents attributes that promote the control of fomites, while red indicates confounding attributes. The size of the text in **(A)** indicates the commonality of themes amongst the participants, as does darker shading in **(B)** [adapted from Schwartz (29). CC BY-SA 4.0].

Two aspects of fomite control were discussed in the qualitative interviews: handling visitor access to the property and hygiene practices used on farm, with Figure 7 representing capability, opportunity, motivations and values for this biosecurity consideration. In this study, hygiene practices related to the practices implemented to minimize the transmission of infectious agents during routine management activities such as vaccination (e.g., changing needles), pregnancy testing (e.g., changing rectal gloves) and artificial insemination (e.g., disinfection of equipment).

Self-direction was the value most positively associated with fomite control. Given that the primary motivation for controlling visitor access was to prevent foreign materials from entering the farm (bioexclusion), proactive behaviors such as placement of signage to alert visitors of movements restrictions and padlocking gates were common themes. This concept extended to other aspects of the farming enterprise like pasture management:

“*– there is a lot of weeds, and who knows what else, where they go to around the area. And a lot of stuff can be invasive as well.”*

–* Participant 11*

“*I padlock all the gates, because we had an incident this year where one of these hobby farms up the road, their cattle got out on the road, and everyone does ‘the right thing' and opens the gate and shoves them in a property.”*

–* Participant 7*

It was well recognized by producers that control of fomites is dependant on the actions of external operators (such as truck drivers, agents, and community members) and so, enforcing restrictions in the early stages of a relationship was seen as crucial to promote consistent behaviors. This assertive behavior, a quality of Self-direction values, was more likely to be expressed if the participant was paying for the external operator's services. Establishment of consistent behaviors in external operators would then foster a habitual relationship that satisfied the value of Security, a theme that was associated with both visitor access and the implementation of on-farm hygiene practices. However, farmers were reluctant to enforce fomite control upon operators with a specific skill set (such as AI technicians and veterinarians) and were more likely to align with Conformist values to maintain amicable relationships and avoid unintended insult:

“*– we respect his position and profession, and knowledge. If he didn't see an issue then we would probably think we didn't need to either.”*

–* Participant 5*

Universalism and Benevolence are values primarily related to self-transcendence, yet their motivational goal regarding fomite control was antithetical. Producers were reluctant to increase the frequency of needle and glove changes during vaccinations and pregnancy testing, respectively, due to Universalist concerns regarding environmental waste from discarded consumables. However, Benevolent values were expressed by some participants in relation to improved animal welfare:

“*[changing the needles more often makes it] easier to go in and less discomfort for the animal”*

–* Participant 7*

There was a generalized resistance to changing needles during vaccination due to the amount of time it would add to existing farm management procedures and impracticality with the current equipment; however, participants indicated that they would adopt the practice in response to a specific disease if there was evidence of efficacy. One participant indicated that transfer of [pesti] virus material *via* needles may be beneficial for immunoprophylaxis, but most producers were more likely to invest resources in alternative biosecurity practices that are likely to be more effective:

“*I mean, hopefully there would be a different way that we can deal with [disease] without having to [change needles].”*

–* Participant 8*

#### 3.4.4. Prophylaxis

**Figure 8:**
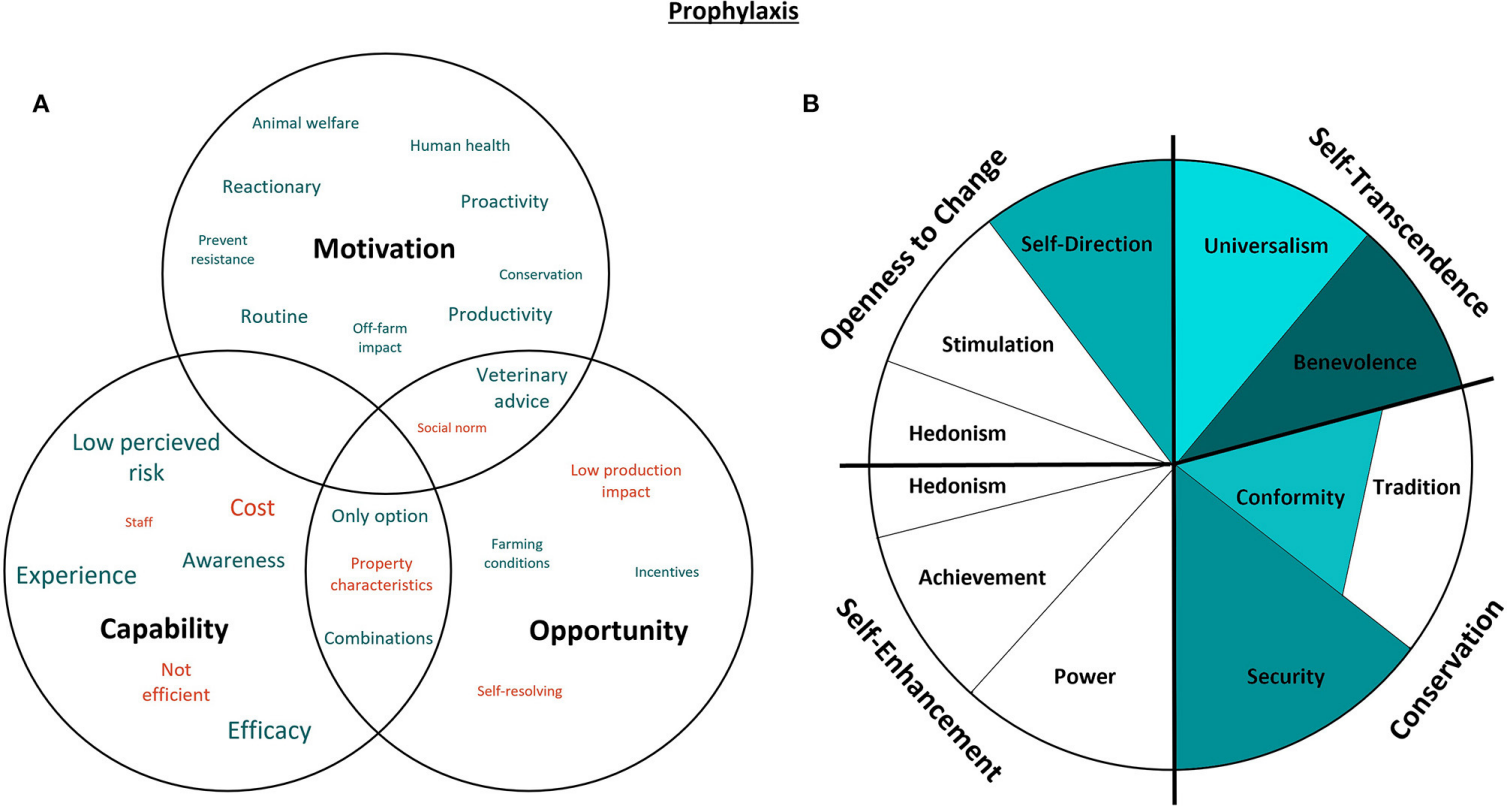
Capability, opportunity, motivations and values associated with the use of prophylaxis in beef cattle. Green represents attributes that promote the use of prophylaxis, while red indicates confounding attributes. The size of the text in the Venn diagram **(A)** indicates commonality of themes amongst the participants, as does darker shading in the value wheel **(B)** [adapted from Schwartz (29). CC BY-SA 4.0].

The interviews highlighted three main types of prophylactic interventions used by Australian beef farmers: chemoprophylaxis (e.g., deworming products, insect repellants, antimicrobials), immunoprophylaxis (e.g., vaccinations) and intentional exposure to disease agents (e.g., strategic PI exposure^6^). Figure 8 represents the capability, opportunity, motivations, and values associated with the use of prophylaxis. One of the primary motivations for prophylactic interventions was to protect the productivity of the enterprise, hence the inclusion of intentional exposure to disease agents in this study.

Benevolence was the most predominant value relating to the use of prophylactic interventions. Preserving the welfare of cattle on farm was associated with all types of prophylaxis and the use of vaccination combinations such as 7-in-1 was specifically mentioned to reduce the number of times that cattle are exposed to needles. All participants who used 7-in-1 instead of the typical 5-in-1 clostridial vaccination combination also did so to protect their employees from zoonoses. Some participants vaccinate against BVDV to protect the buyers of their cattle, with concerns about the off-farm impact of the virus in feedlots or reproductive systems. Most participants did not vaccinate their cattle for BVDV because they perceive the risk of disease exposure to be low; however, the majority of those that did vaccinate against BVDV did so in reaction to a loss in production or awareness of active infection:

“*We've had two or three cases of pesti[virus] earlier on and that led to a number of abortions. We now vaccinate for pesti[virus] and we've probably got a naïve herd.”*

–* Participant 10*

This independent thought, an attribute associated with the value of Self-direction, was a principal competent of prophylactic use. All but one participant was reluctant to use strategic PI exposure and the main reason for this was that the practice was either viewed as inefficient (difficult to confirm, source and maintain the infected animal) or that it was likely not as efficacious as providing immunoprophylaxis as vaccination. The interview participant who used strategic PI exposure instead of vaccination did so for the same reasons:

“*I don't know if I'd go back to [pestivirus] vaccinations. If you went back to vaccination then I think you've got to do the whole herd, so. The cost of doing that and the management of doing it, you know… we're scaled over a wide location.”*

–* Participant 9*

The decision to use prophylactics was also influenced by Security values relating to trust in veterinary advice. Many participants indicated that they vaccinate their bulls for campylobacteriosis (vibriosis) proactively based on trusted advice from their veterinarian, even if they had not experienced any drop in fertility. Prolonged trust in the necessity of the practice results in a routine which further satisfies values of Security. Widespread habitual use of prophylactic interventions resulted in subsequent Conformist adoption from those new to the industry:

“*We've always vaccinated against clostridial diseases, and we've given the bulls vibriosis vaccines without ever having had an outbreak of any of those diseases, that we know of.”*

–* Participant 5*

Universalist values were associated with the adoption of strategic drenching programs for internal parasites (as opposed to annual blanket drenching) amongst some participants who were concerned about environmental impact. One participant adopted strategic drenching to limit impacts on the dung beetle population and another indicated that they focus on prophylaxis to reduce antimicrobial use. As with other prophylactic interventions, strategic drenching programs were primarily adopted in response to persistence of clinical signs. Participants were less likely to implement chemoprophylaxis if the disease agent resulted in minimal production losses or was self-resolving, which was the unanimous approach for cattle lice prevention.

“*I'm saving the world on my own. We know that we don't want more penicillins used than it needs to be used, so don't use it.”*

–* Participant 6*

### 3.5. The cultural significance of biosecurity values

**Figure 9:**
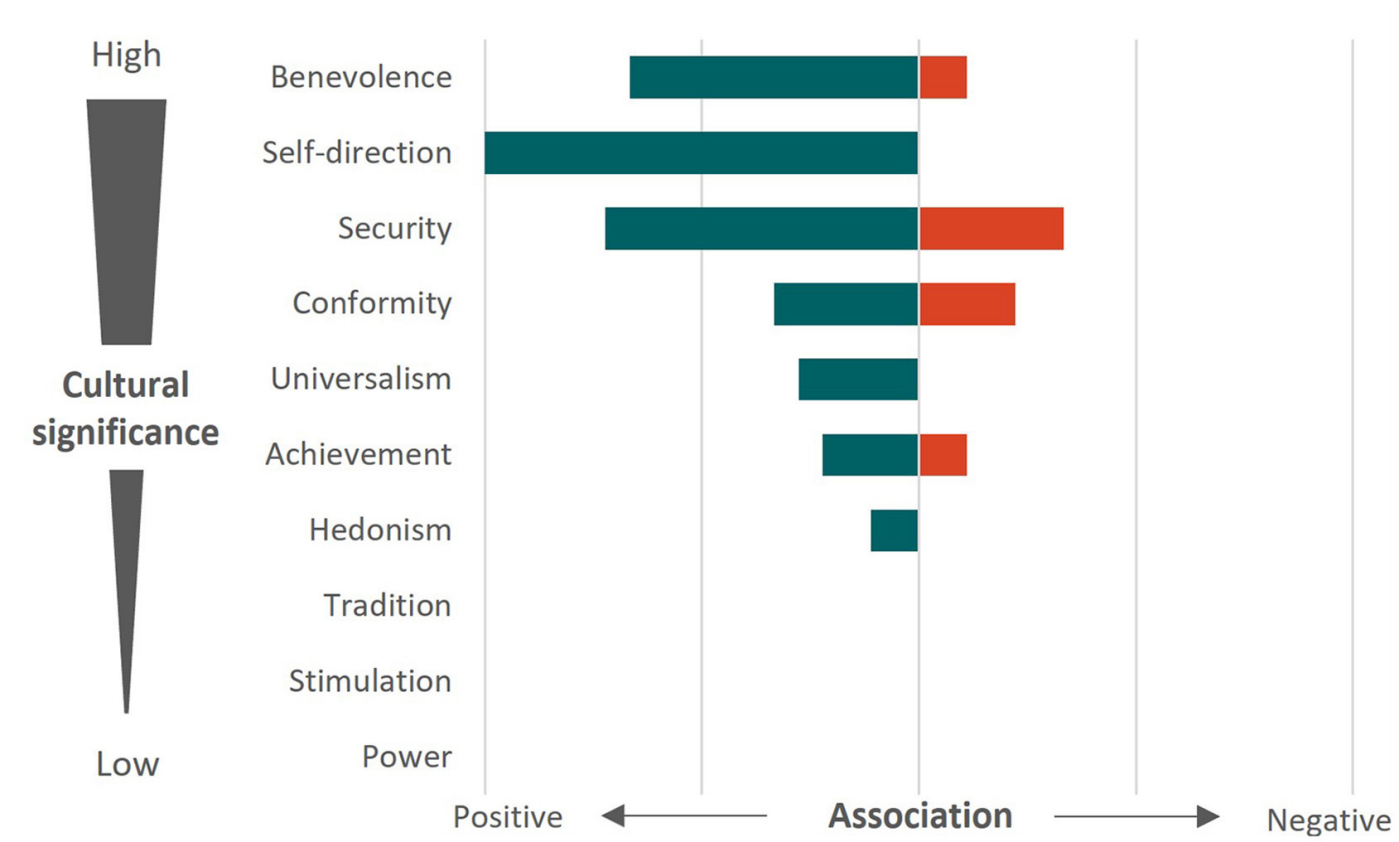
Comparative occurrence of values expressed by Australian beef cattle farmers for the four primary biosecurity behaviors elicited from semi-structured interviews. Biosecuirty behaviors included isolating cattle, protecting farm boundaries, fomite control and prophylaxis. Green and red represent positive and negative associations, respectively.

Seven of the ten basic human values were found to relate to positive biosecurity behaviors based on participant responses and were generally expressed in order of cultural importance (Figure 9). Self-direction, Security and Benevolence were the three values that were most commonly related to positive behaviors, which are ranked second, third and first in order of cultural importance, respectively. Tradition, Stimulation and Power, ranked as the least culturally important values, were not expressed in any of the themes relating to on-farm biosecurity. Security and Conformity were the values most commonly found to prevent biosecurity behaviors, followed by Benevolence and Achievement.

## 4. Discussion

There are several studies that examine the socio-psychological factors that influence the biosecurity behaviors of farmers across a wide range of countries, including Canada (8), Britain (23, 35, 41), New Zealand (22, 42) and Australia (9, 33). Between these countries, there are similarities and differences that might arise due to production system types, mandatory requirements, and access to resources. However, the influence of held (or moral) values on human behavior is a factor that is consistently referenced across the socio-epidemiological literature and so in this study, we used values to characterize the biosecurity behaviors of Australian beef cattle farmers. Schwartz (29) suggests that every individual holds numerous values that transcend specific situations, yet the relative importance of these values will change depending on context. Throughout this study we determined that seven of the ten values were exhibited by farmers across the four biosecurity categories; however, these values changed depending on the practice. Schwartz (29) also describes the “pan-cultural baseline of value priorities,” which describes the order in which values align with the demands and requirements of society to promote cooperation and group prosperity. We would further suggest that the expression of values that align with cultural priorities, in the context of national disease management, would serve to facilitate collaborative efforts to prevent the establishment of emerging diseases and reduce the impact of endemic diseases (the greater good). Fortunately, the primary values expressed by farmers during discussions relating to on-farm biosecurity (Benevolence, Self-direction and Security) were ranked first, third, and fourth as values of cultural importance by Schwartz (29). Furthermore, the values that are of lowest cultural importance (Power, Stimulation and Tradition) were not found to be related to the biosecurity behaviors of Australian beef farmers. If our goal as a collective is to preserve the health of the national cattle herd, these findings support the use of strategies which consider the held values that align with biosecurity behaviors and suggest that these strategies are most likely to result in positive widespread behavior change.

Self-direction and Benevolence were the two values that were consistently identified as positive contributors to on-farm biosecurity. Self-direction values are characterized by independent thought and action (29). In the context of on-farm biosecurity, Self-direction was manifested as independent farmer decision-making, based on their knowledge of disease impact, their perceived risk of the disease affecting their herd, and the perceived efficacy of the practice. We found that famers are generally willing to deviate from normal practice if they determine that there is a need to do so, a view which is expressed in several other studies addressing on-farm biosecurity (8, 43, 44). Previous experiences with the diseases and biosecurity practices were also primary factors that affected the adoption of positive biosecurity behaviors. An example that arose from the interviews was the similarities between the decision to vaccinate for BVDV vs. the use of strategic PI exposure. While most Australian veterinarians (and several of the interview participants) might disapprove of the use of strategic PI exposure (45), the decision to implement the practice was a calculated decision based on this participants' interpretation of potential losses in production, the efficacy of PI transmission resulting in immunity, and previous negative experiences with vaccination. Likewise, the decision to vaccinate was the result of an assessment of potential production losses, the certainty of vaccination efficacy and negative experiences with the disease. These examples describe the same motivation to manage BVDV through immunoprophylaxis; however, the decision outcome is different.

Benevolence, the other predominant value identified to be related to on-farm biosecurity behaviors, emphasizes the voluntary concern for the welfare of those with whom one is in frequent personal contact (29). In the context of on-farm biosecurity, Benevolence was expressed primarily as a concern for cattle welfare, the health and welfare of employees, and to a lesser extent the impact of their enterprise on other farmers in the beef industry. Based on this study, biosecurity decision-making is relatively self-transcendent, regardless of whether farmers are directly aware of this. The ubiquity of Self-direction and Benevolence values in biosecurity behavior provides an opportunity for industry-wide improvement. We acknowledge that cost-benefit and productivity are repeatably cited as reasons for on-farm decision making by our interview participants and in the literature (12, 22, 46). However, any remaining apprehension to adopt and implement the biosecurity standards desired by government and industry might be mitigated with better access to knowledge about the risk of endemic disease exposure, the underlying impact of endemic diseases on cattle health, and how biosecurity practices can impact these factors. The relationship between knowledge and biosecurity implementation is not a new concept in this field (7, 8, 12, 21, 47); however, in this study we argue that the type of knowledge that most influences values of Self-direction and Benevolence is awareness (8, 21). That is, awareness of disease prevalence, awareness of the subclinical impact of disease and awareness of how relative on-farm interventions affect the welfare of cattle and the wider industry. Regional prevalence surveys to inform producers of the current risk of priority diseases for the industry, as well as research that better quantifies the direct impact of biosecurity practices on individual cattle health both on-farm and when cattle leave the farm, could facilitate communication about the benefits of biosecurity between field veterinarians and cattle producers.

Values relating to Security were positively associated with all aspects of on-farm biosecurity identified in this study except for isolation of cattle. Security refers to the safety, harmony and stability of self, society, and relationships (29). In the context of biosecurity, we found that Security was represented by trust in family, veterinarians, technicians, and others that work in the beef farming industry, as well as adherence to routine farming behaviors. Security is usually considered a conservative value that contradicts the concept of change; however, in the context of agriculture, we argue that Security is integral. If Self-direction and Benevolence create the change, Security acts to ensconce desired behaviors and ensure their continued use by producers in the absence of active disease outbreaks. The relationship between farmer and veterinarian is consistently mentioned as crucial for biosecurity uptake and adoption (7, 19, 21, 23, 30). There were several examples in the interviews that indicated a reliance on veterinary advice when deciding to vaccinate for vibriosis. In some cases, vaccination became a routine practice, regardless of whether there was an active risk to the herd. Additionally, in the current study veterinarians were very influential in how to manage BVDV following an outbreak. However, biosecurity behaviors might deviate from those desired by government and industry if trusted private veterinary advice does not align with industry priorities. Again, the use of strategic PI exposure is an example. Regional and national control of BVDV is hindered by strategic PI exposure and therefore, it is in the beef industry's best interest to discourage the practice. However, on an individual level, some veterinarians discuss the practice with farmers to improve trust and maintain the relationship (45). While this study has illustrated that some producers may initially find strategic PI exposure attractive, validating the purposeful exposure of cattle contributes to word-of-mouth adoption between farmers which can be of further detriment to the industry. This is a reminder about the importance of consistent messaging between those in the animal health industry toward a common goal.

Security values were also found to be antithetical to desired isolation behaviors. Participants in this study exhibited apathy toward the health status of introduced bulls, given their trust in the practices of their supplier. This trust was fostered by consistent relationships, and given that Security values are conservative, we acknowledge that industry interventions are unlikely to change the isolation practices of those beef farmers who maintain a trusting relationship with their bull supplier. The existence of these relationships should not be viewed as negative. While the beef farmers in this study indicate that they purchase bulls from “reputable breeders,” there is no evidence of the quality of biosecurity on beef stud farms to validate this assumption. In the case that stud farm biosecurity protocols are lacking, we suggest that interventions targeting cattle supply chains such as stud farms would optimize veterinary resources with benefit to the wider beef farming community.

The values of Security are very closely related to those of Conformity. Established relationships and the desire to feel secure in a group can result in Conformity of biosecurity behaviors. We found that this was beneficial if group practices aligned with desired biosecurity behaviors. However, Conformity was mostly found to be antagonistic to the development of good biosecurity. According to Schwartz (29), Conformity values describe the self-restraint of actions, inclinations and impulses that are likely to upset or violate social norms. They are self-protective and, in this study, we found that they were typically associated with reluctance to implement a biosecurity practice so as to not offend those within the farmer's social circle. This was especially the case with the erection of double fences and the enforcement of visitor hygiene. Note that Tradition, a value that shares similar motivations with Conformity, was not identified as an attribute in any of the biosecurity behaviors explored in this study. Tradition was interpreted as strict and devout adherence to a behavior and is the antithesis of Self-direction (29). The fact that traits were identified as Conformist and not Traditional indicates that, at least in the context of farm biosecurity, behaviors may be subject to change. However, Conformity also contradicts Self-direction and so the extent to which these values affect the adoption of desired biosecurity behaviors is dependent on the traits of the individual. Given this bipolar relationship, we suggest that identification of these traits in individuals might expedite the widespread adoption of good biosecurity principles. Social norms and the perceived opinions of family, neighbors, and others with whom Australian farmers have a close personal relationship have been shown to influence their perceived need for adherence to animal health standards (20, 48). To capitalize on this relationship, the Australian Government has profiled several “biosecurity champions” to promote good biosecurity principles in specific agricultural industries (49). We believe that investment in biosecurity champions will only be effective if the target audience values Conformity higher than Self-direction. Alternatively, those that value Self-direction will play an important role as biosecurity champions. This is especially important in regions with a high number of part-time and smallholder farms. Industry and government stakeholders who aspire to achieve widespread adoption of on-farm biosecurity should focus their resources on ensuring that actors with a high level of Self-direction are adhering to desired biosecurity behaviors, while nurturing farmer networks to facilitate diffusion of these behaviors to the wider farming population with Conformist values.

Apart from Self-direction, most of the values associated with biosecurity behaviors are social. Contrary to this, we found that the values associated with the underlying decision to farm beef cattle lie on the “personal” (left) side of the values-wheel. Hedonism was the predominant value attributed to cattle farming, which relates to the enjoyment and pleasure experienced by the farmer. That the decision to farm cattle is characterized by personal values, affirms the notion of the “farming identity” (50). While it may seem that this has no direct relevance to biosecurity behaviors, beef farmers are consistently exposed to external factors, such as climate change, environmental hardship, volatile consumer preferences and animal welfare activism, that can challenge this identity (1, 51–53). We should acknowledge that these competing challenges might reduce the capacity for producers to engage with new biosecurity behaviors. Overcoming these challenges might be seen as the personification of Achievement, which was identified as a component of cattle farming and closely related to Hedonism. Prestige, social status, and authority were not themes relating to farming or endemic disease management, and thus attributes relating to Power was not identified in this study. However, Power may be a factor in behaviors relating to farmer identity and adherence to biosecurity during a national disease outbreak (6). We identified that Self-direction is a principal value associated with the need to farm cattle and according to Schwartz (29), Hedonism and Self-direction promote self-expansion and growth; traits which foster resilience and openness to change in times of hardship and challenges to farming identity. In 2013 Gerber et al. (51) reported that the beef cattle industry accounts for just under half of all global carbon emissions caused by the livestock sector, resulting in a social condemnation toward beef farming practices. This social pressure provides new opportunities to present biosecurity strategies as concurrent solutions to these external factors. For example, several participants interviewed in this study use shelter belts, both as a biosecurity measure and to offset the carbon emissions produced by farming beef cattle. Therefore, we suggest aligning biosecurity recommendations with the social issues that impact beef farming to improve the incentive to adopt new practices.

Just as factors external to the farm might influence a producer's perception of disease management practices, so too can their opinion of the disease (19, 23). Farmers are more likely to implement additional measures if they deem the disease to be a priority (8). As animal health professionals, we categorize diseases to better describe how they might move through a cattle population and to what extent the disease can be managed. For example, a disease like “woody tongue” (*Actinobacillus lignieresii*) is considered opportunistic, affecting animals sporadically only under specific conditions, and therefore receives infrequent attention from animal health authorities when compared to an endemic disease like pestivirus. However, in this study we found that farmers had an understanding of what constitutes an “endemic disease” that was inconsistent with academic definitions, similar to their reported understanding of emergency animal diseases (30). Some producers were likely to perceive opportunistic or environmental diseases like foot abscesses as endemic diseases if they witness the same disease event on a regular basis. Alternatively, labeling a disease as “endemic” might desensitize farmers to the potential impact of the disease, as the term is sometimes used synonymously with “mild” as made evident by recent events in human health regarding SARS-CoV-2 (54). These misinterpretations serve as reminders to be conscious of the terminology used in disease education campaigns targeting farmers. There is also a recency component to producers' risk perception of a specific disease, which was apparent in the interviews by the repeated mentions of lumpy skin disease which was detected in countries close to Australia's international borders at the time of this study. Several of the interviewed participants indicated that low-grade, yet problematic diseases are sometimes overshadowed by other farming priorities and that consistent reminders (like participation in this study) are appreciated. While this is achieved more broadly in Australia with requirements like the LPA biosecurity plan (3), we suggest that farmers are more likely to put these biosecurity concepts into practice if education is directed toward individual priority diseases.

The foundation of this study was based predominantly on concepts and work by Schwartz (29). The values described by Schwartz (29) are a widely accepted representation of the basic values inherent to all human individuals (55, 56). These values are traditionally used as the basis for research describing and comparing the value systems of specific demographics and cultures (40, 55). However, in this current study, we used the ten values to compartmentalize and explain different biosecurity behaviors in the beef industry, rather than as a literal representation of the actual values of Australian beef farmers. It was not within the scope of this study to create a values ‘profile' of the Australian beef farmer and future research could consider adopting the Schwartz Value Survey (40) or the Portrait Values Questionnaire (56) to determine the actual held values of the Australian beef producer. Likewise, the order to which values are culturally important is largely consistent between different groups; however, the order that we have used to demonstrate the cultural significance of farmer values is not specific to the beef industry. Despite these shortcomings, we argue that our approach is useful for industry stakeholders when deciding how to design extension materials. The methods that we have employed throughout this study reflect the held values and the traits of our interview participants. A participant might not consider Conformity to be one of their held values, yet the same participant may exhibit traits of Conformity. Just as reported preferences are just as important to economists as revealed preferences (57), traits should be considered in tandem with values when guiding behavior change. We also acknowledge that the compartmentalization of participant behaviors into value categories was largely based on the primary authors interpretation of the interview data and how it related to accepted definitions of the ten basic human values. This is the nature of qualitative research; however, to improve transparency and repeatability the authors have described how they characterized each of the basic values in relation to biosecurity behavior (Table 1). Lastly, with our reliance on convenience sampling, it is likely that the results of this study are biased toward a particular type of farmer; that is, farmers with altruistic tendencies and the initiative to volunteer for an online survey and a 60–90-min interview. We acknowledge that this might contribute to the overrepresentation of Self-direction and Benevolent values in our findings and do not suggest that they reflect the wider beef farming industry. However, this study does provide a foundation for researchers and extension officers to consider held values when designing intervention strategies to improve on-farm biosecurity behaviors.

## 5. Conclusion

In conclusion, this study found that Benevolence and Self-direction are the principal values associated with positive biosecurity behaviors in Australian beef farming; values ascribed to self-transcendence and an openness to change in the held values literature. Security values were also an important component of biosecurity behavior; however, we also found that farmers were unlikely to engage in isolation practices if secure relationships existed with cattle suppliers. Overall, the values identified in this study were those most significant for cultural prosperity, which suggests that collaborative disease management efforts between government, industry and farmers are likely to succeed; however, stakeholders should be wary about the use of approaches (such as mandates) that might subvert or challenge the values of Self-direction. Future interventions would benefit from the quantification of priority disease transmission and impact on cattle health to help guide biosecurity decision-making and nurture Self-direction and Benevolent tendencies.

## Data availability statement

The datasets presented in this article are not readily available because they are restricted by human ethics and will require an approval process prior to distribution. Requests to access the datasets should be directed to jfountain@csu.edu.au.

## Ethics statement

The studies involving human participants were reviewed and approved by the Human Research Ethics Committee of Charles Sturt University. The ethics committee waived the requirement of written informed consent for participation.

## Author contributions

The study was designed by JF, VJB, MH-J, and JM as part of a PhD project. JF was responsible for recruitment, conducting interviews, and analyzing the data, with assistance from VJB, MH-J, and JM. All authors read and approved the final manuscript.

## Footnotes

^1^Their perception of how farmers deal with issues.

^2^Their perception of what is approved by other people.

^3^Grandparents and parents were beef cattle farmers.

^4^A person not born into farming.

^5^Parents were beef cattle farmers.

^6^Strategic PI exposure refers to intentional exposure to an animal persistently infected with bovine viral diarrhoea virus.
