# Supplementary material for: Understanding biosecurity behaviors of Australian beef cattle farmers using the ten basic human values framework
Source: Front Vet Sci. 2023 Feb 27;10:1072929. doi: 10.3389/fvets.2023.1072929 (PMC10010389; doi:10.3389/fvets.2023.1072929)

Supplementary Material

# Supplementary Data

## Semi-qualitative script for project interviews

Prologue – Participant consent

1. Do you provide your verbal consent to be recorded in this interview, with the knowledge that you will not be identifiable in the final version of the transcript?

Part A – Endemic disease and general disease control discussion

1. Why do you farm beef cattle?

Now I’d like to discuss your experiences and opinions about endemic diseases, more specifically infectious endemic diseases.

1. How do you define: infectious endemic disease?

- Our definition: An infectious organism (parasite, virus, bacteria) that is constantly maintained at a baseline level in a geographic area without external inputs.
- (If participant has a different opinion/definition of what an infectious endemic disease is to ours, correct and provide an example of what it is NOT, e.g. bloat, milk fever, etc.; to avoid prompting participant to think about specific types of infectious disease)

1. According to your survey responses, you’ve been farming beef cattle now for #insert timeframe here#; in that time what experiences have you had with infectious endemic diseases in your beef herd?

Prompting comments/questions – if not raised by the producer:

- How long ago did the experience occur (if not ongoing)?
- When did you realize that ‘disease’ was a problem?
- Are you still concerned about ‘the disease’/is ‘the disease’ still a problem for you?
- How did that (the impact of the disease) feel/how did that (the impact of the disease) make you feel?

1. What actions did you take to control or eliminate ‘diseases in Q4’?

- Prompting comments/questions – if not raised by the producer:
- What prompted you to take action?
- Why didn’t you attempt to control/eliminate ‘the disease?’

1. Are there any infectious endemic diseases that you didn’t encounter in your own herd that you were particularly concerned about?

Prompting comments/questions – if not raised by the producer:

- Why are you concerned about ‘the disease?’
- What steps (if any) have you put in place to reduce the risk of introducing the disease to your cattle?
- How does that relate to your relationship with the other farms in your area?
- Are you concerned about exotic or emerging diseases?

1. Are there any infectious endemic diseases that you’re not concerned about?

Prompting comments/questions – if not raised by the producer:

- Why aren’t you concerned about ‘the disease?’
- When would you be worried about ‘the disease?’

Part B – Specific biosecurity practice discussion

(Note that this section might be undertaken alongside Part A depending on their responses)

1. We’ve discussed several strategies that you’ve used to prevent/manage ‘Disease X’. What other strategies have you heard of that can be used to prevent/manage infectious endemic diseases?

Prompting comments/questions – if not raised by the producer:

- What’s your personal experience with ‘the strategy?’
  - If used previously, why don’t you use the strategy now?
- In what circumstance(s) might consider using ‘the strategy?’
  - If none, why not?

1. We’ve touched on a few of the reasons why you did/did not implement ‘the strategy’ to manage infectious endemic diseases. What are the main things that influence your decision to implement a practice on farm?
2. What factors in your farming operation take priority over prevention or management of diseases in your cattle?

- If yes, why do these things take priority?

Part C – Survey discussion

At this point I’d like to discuss the survey that you participated in last year. Before we get into the finer details:

1. What do you have to say about the survey in general?

What we wanted to do with the survey was collect information about how you, as a beef producer, prioritize certain aspects of disease management in cattle. Before I get into how we used that information I’d like to discuss some of your responses.

1. You might remember in that last section of the survey we used a number of criteria to reflect the performance of those theoretical disease management strategies labelled A to P. Those criteria included:
   - The ability to prevent establishment of the disease in the herd.
   - The total number of infected cattle, abortions, calf deaths and underweight cattle sold to feedlot once the disease becomes established.
   - The total number of sick cattle sold to feedlot
   - And the annual input cost of the management strategy.

Of those criteria, which ones would you generally prioritize over the other?

1. Apart from those seven criteria, what other criteria would you have liked to have had available before deciding on the performance of each strategy?

(Additional question if answer to Q10 does not correlate with our findings)

1. When looking at your responses, we found that you tended to score scenarios that reduced the on-farm effect of bovine pestivirus highly; in other words, those with the lowest infection, abortion and calf mortality rates with some credence towards a lower risk of disease introduction.

What is your opinion on that finding?

Part D – Survey outcome discussion

You might be interested to know that the range of values that we used to populate those theoretical criteria came from a simulation model that we’ve developed looking at bovine pestivirus in the Australian beef industry:

1. Have you heard of bovine pestivirus?
   - If so, what can you tell me about bovine pestivirus?
2. What’s your experience with bovine pestivirus in your cattle?

See question (3) for prompting questions.

1. What strategies are you aware of that might help to prevent bovine pestivirus introduction or spread in a beef herd?
2. What would you define as a ‘biosecurity practice’?

- Would you consider any of those strategies that you’ve just mentioned a biosecurity practice?

1. What is your experience with using (if not mentioned in Q15)?
   - Isolation of purchased animals
   - Replacing needles and pregnancy testing gloves during vaccination
   - Double fencing boundaries with neighboring producers
   - Vaccination against bovine pestivirus
   - Have you ever introduced bovine pestivirus intentionally?

So, what we’ve done with your responses is pool them together with the responses from all of the other participants to give us a general idea about the priorities of beef producers as a whole.

We then used this information to determine which combination of those five on-farm biosecurity practices we would recommend to reduce the negative impacts of bovine pestivirus on a self-replacing beef herd, based on the priorities of all survey participants.

1. Before I reveal what we’ve found, in your opinion and based on your experience, which of these practices would you use to prevent BVDV?
   - Why?

We found that the most recommended strategies, based on all of the participant priorities, was any strategy that included double-fencing boundaries with the neighboring farm in combination with vaccination against bovine pestivirus

1. What is your opinion with that finding?
   - Why do you agree/why don’t you agree?

Part E - Conclusion

1. Before we finish up, is there anything else that you’d like to add to this discussion today?

## Participant information statement for interviews


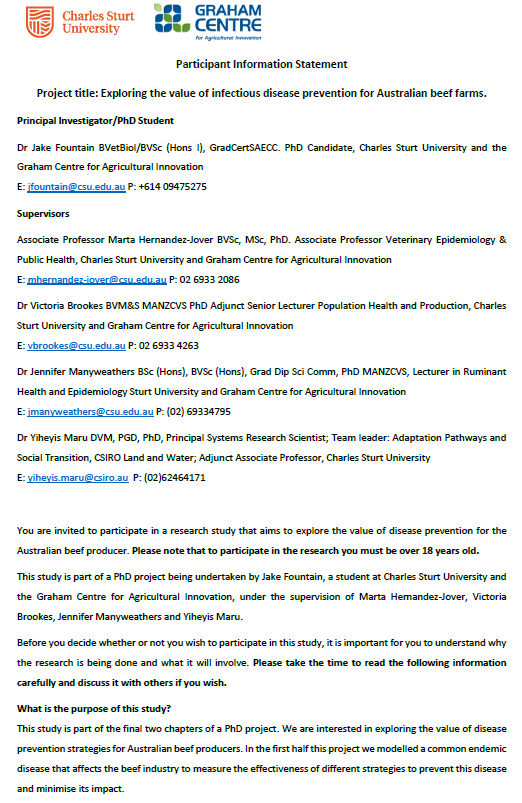


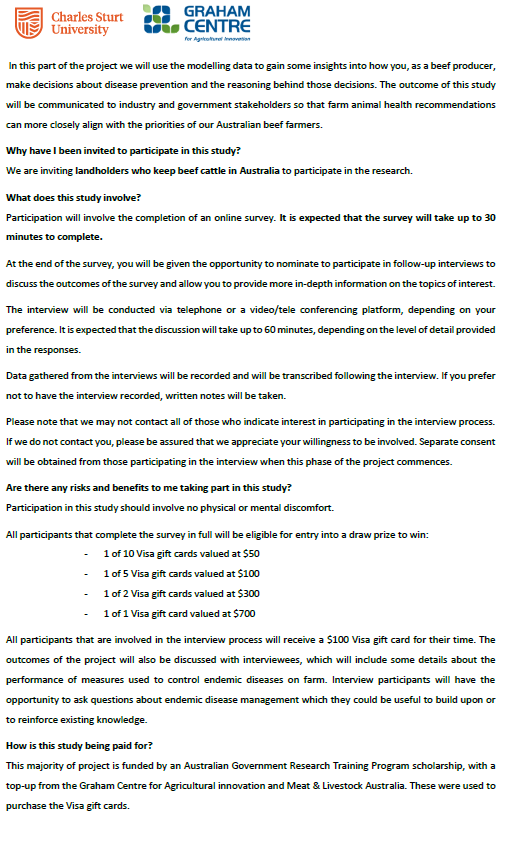


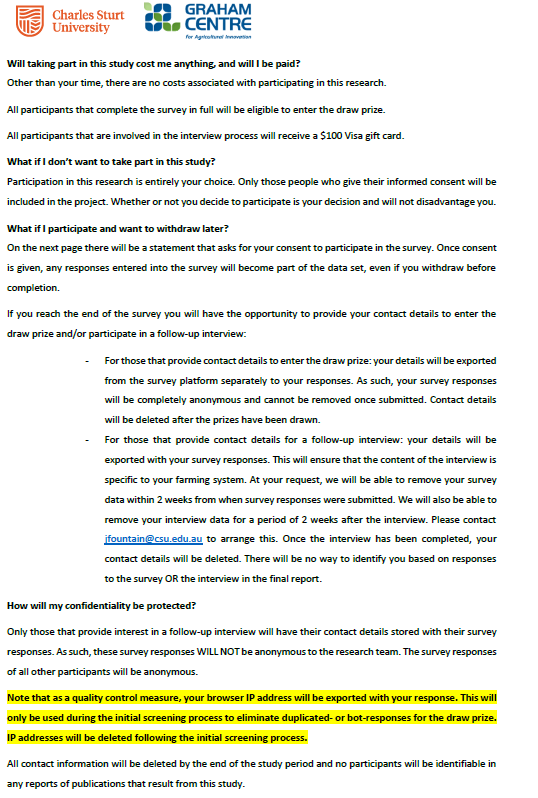


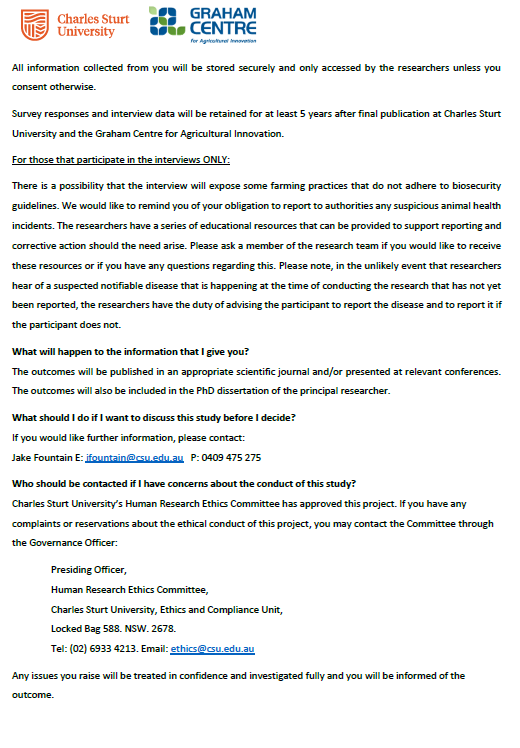

Supplement: Supplementary file 1 [file Data_Sheet_1.docx]
